# Transarterial chemoembolization with PD-(L)1 inhibitors plus molecular targeted therapies for hepatocellular carcinoma (CHANCE001)

**DOI:** 10.1038/s41392-022-01235-0

**Published:** 2023-02-08

**Authors:** Hai-Dong Zhu, Hai-Liang Li, Ming-Sheng Huang, Wei-Zhu Yang, Guo-Wen Yin, Bin-Yan Zhong, Jun-Hui Sun, Zhi-Cheng Jin, Jian-Jian Chen, Nai-Jian Ge, Wen-Bin Ding, Wen-Hui Li, Jin-Hua Huang, Wei Mu, Shan-Zhi Gu, Jia-Ping Li, Hui Zhao, Shu-Wei Wen, Yan-Ming Lei, Yu-Sheng Song, Chun-Wang Yuan, Wei-Dong Wang, Ming Huang, Wei Zhao, Jian-Bing Wu, Song Wang, Xu Zhu, Jian-Jun Han, Wei-Xin Ren, Zai-Ming Lu, Wen-Ge Xing, Yong Fan, Hai-Lan Lin, Zi-Shu Zhang, Guo-Hui Xu, Wen-Hao Hu, Qiang Tu, Hong-Ying Su, Chuan-Sheng Zheng, Yong Chen, Xu-Ya Zhao, Zhu-Ting Fang, Qi Wang, Jin-Wei Zhao, Ai-Bing Xu, Jian Xu, Qing-Hua Wu, Huan-Zhang Niu, Jian Wang, Feng Dai, Dui-Ping Feng, Qing-Dong Li, Rong-Shu Shi, Jia-Rui Li, Guang Yang, Hai-Bin Shi, Jian-Song Ji, Yu-E Liu, Zheng Cai, Po Yang, Yang Zhao, Xiao-Li Zhu, Li-Gong Lu, Gao-Jun Teng, for the CHANCE001 Investigators

**Affiliations:** 1 Center of Interventional Radiology & Vascular Surgery, Department of Radiology, Zhongda Hospital, Medical School, Southeast University, Nanjing, 210009 China; 2 Department of Minimally invasive Intervention, The Affiliated Cancer Hospital of Zhengzhou University, Zhengzhou, 450008 China; 3 Department of Interventional Radiology, the Third Affiliated Hospital, Sun Yat-Sen University, Guangzhou, 510630 China; 4 Department of Interventional Radiology, Union Hospital of Fujian Medical University, Fuzhou, 350001 China; 5 Department of Interventional Radiology, Jiangsu Cancer Hospital & Jiangsu Institute of Cancer Research & The Affiliated Cancer Hospital of Nanjing Medical University, Nanjing, 210009 China; 6 Department of Interventional Radiology, The First Affiliated Hospital of Soochow University, Soochow University, Suzhou, 215006 China; 7 Hepatobiliary and Pancreatic Interventional Treatment Center, Division of Hepatobiliary and Pancreatic Surgery, The First Affiliated Hospital, Zhejiang University School of Medicine, Hangzhou, 310003 China; 8 Department of Interventional Radiology, Eastern Hospital of Hepatobiliary Surgery, Navy Medical University (Second Military Medical University), Shanghai, 200438 China; 9 Department of Interventional Radiology, Nantong First People’s Hospital, Nantong, 226001 China; 10 Department of Interventional Radiology, Yancheng Third People’s Hospital, Yancheng, 224008 China; 11 Department of Minimally Invasive Interventional Therapy, Sun Yat-Sen University Cancer Center, Guangzhou, 510060 China; 12 Department of Vascular Surgery, Southwest Hospital, Third Military Medical University (Army Medical University), Chongqing, 400038 China; 13 Department of Interventional Radiology, Hunan Cancer Hospital, Changsha, 410031 China; 14 Department of Interventional Oncology, The First Affiliated Hospital of Sun Yat-sen University, Guangzhou, 510080 China; 15 Department of Interventional Radiology, The Hospital of Nantong University, Nantong, 226001 China; 16 Department of Interventional Therapy, Shanxi Tumor Hospital, Taiyuan, 030001 China; 17 Department of Interventional Radiology, Tibet Autonomous Region People’s Hospital, Lhasa, 850000 China; 18 Department of Interventional Radiology, Ganzhou People’s Hospital, Ganzhou, 341000 China; 19 Center of Interventional Oncology and Liver Diseases, Beijing Youan Hospital, Capital Medical University, Beijing, 100069 China; 20 Department of Interventional Radiology, The Affiliated Wuxi People’s Hospital of Nanjing Medical University, Wuxi, 214023 China; 21 Department of Minimally Invasive Interventional Therapy, Yunnan Tumor Hospital, The Third Affiliated Hospital of Kunming Medical University, Kunming, 650118 China; 22 Department of Radiology, First Affiliated Hospital of Kunming Medical University, Kunming, 650032 China; 23 Department of Oncology, The Second Affiliated Hospital of Nanchang University, Nanchang, 330006 China; 24 Department of Interventional Radiology, Affiliated Hospital of Qingdao University, Qingdao, 266000 China; 25 Department of Interventional Therapy, Peking University Cancer Hospital and Institute, Key Laboratory of Carcinogenesis and Translational Research (Ministry of Education), Beijing, 100142 China; 26 Department of Interventional Radiology, Affiliated Cancer Hospital of Shandong First Medical University, Jinan, 250117 China; 27 Interventional Therapy Center, The first Affiliated Hospital of Xinjiang Medical University, Urumqi, 830011 China; 28 Department of Radiology, Shengjing Hospital of China Medical University, Shenyang, 830011 China; 29 Department of Interventional Oncology, Tianjin Medical University Cancer Hospital, Tianjin, 300060 China; 30 Department of Radiology, Tianjin Medical University General Hospital, Tianjin, 300052 China; 31 Department of Tumor Interventional Therapy, Fujian Cancer Hospital, Fuzhou, 350014 China; 32 Department of Radiology, The Second Xiangya Hospital, Changsha, 410011 China; 33 Department of Interventional Radiology, Sichuan Cancer Hospital and Institute, Chengdu, 610041 China; 34 Department of Interventional Radiology, First Affiliated Hospital of Wenzhou Medical University, Wenzhou, 325000 China; 35 Department of Hepatobiliary Oncology Surgery, Department of Interventional Oncology, Jiangxi Cancer Hospital of Nanchang University, Nanchang, 330029 China; 36 Department of Interventional Radiology, The First Hospital of China Medical University, Shenyang, 110001 China; 37 Department of Radiology, Union Hospital, Tongji Medical College, Huazhong University of Science and Technology, Wuhan, 110001 China; 38 Department of Interventional Radiology, General hospital of Ningxia Medical University, Yinchuan, 110001 China; 39 Department of Interventional Radiology, Guizhou Cancer Hospital, Guiyang, 550000 China; 40 Department of Interventional Radiology, Fujian Provincial Hospital, Shengli Clinical Medical College of Fujian Medical University, Fuzhou, 350001 China; 41 Department of Interventional Radiology, Third Affiliated Hospital of Soochow University, Changzhou First Hospital, Changzhou, 213004 China; 42 Department of Interventional and Vascular Surgery, The Affiliated Changzhou No. 2 People’s Hospital of Nanjing Medical University, Changzhou, 213003 China; 43 Department of Interventional Therapy, Nantong Tumor Hospital, Nantong, 226006 China; 44 Department of Interventional Therapy, Jinling Hospital, School of Medicine, Nanjing University, Nanjing, 210002 China; 45 Department of Interventional Radiology, Affiliated Hospital of Jiangnan University, Wuxi, 214122 China; 46 Department of Interventional Radiology, The First Affiliated Hospital, and College of Clinical Medicine of Henan University of Science and Technology, Luoyang, 471003 China; 47 Department of Interventional Radiology and Vascular Surgery, Peking University First Hospital, Beijing, 100034 China; 48 Department of Interventional Radiology, The Second Hospital of Nanjing, Nanjing, 210000 China; 49 Department of Oncology and Vascular Intervention, First Hospital of Shanxi Medical University, Taiyuan, 030001 China; 50 Vascular and Interventional Department, Chongqing University Cancer Hospital, Chongqing, 400000 China; 51 Department of Interventional Radiology, The Affiliated Hospital of Zunyi Medical College, Zunyi, 563000 China; 52 Department of Interventional Therapy, The First Hospital of Jilin University, Changchun, 130000 China; 53 Department of Radiology, The Fourth Hospital of Hebei Medical University, Shijiazhuang, China; 54 Department of Interventional Radiology, The First Affiliated Hospital of Nanjing Medical University, Nanjing, 210029 China; 55 Department of Radiology, Key Laboratory of Imaging Diagnosis and Minimally Invasive Intervention Research, School of Medicine, Lishui Hospital of Zhejiang University, Lishui, 323000 China; 56 Department of Interventional Radiology, Shanxi Provincial People’s Hospital, Taiyuan, 030012 China; 57 Department of Interventional Medicine, The Second Affiliated Hospital of Zunyi Medical University, Zunyi, 563000 China; 58 Department of Interventional & Vascular Surgery, The Fourth Hospital of Harbin Medical University, Harbin, 150001 China; 59 Department of Biostatistics, Nanjing Medical University, Nanjing, 211166 China; 60 Zhuhai Interventional Medical Center, Zhuhai Precision Medical Center, Zhuhai People’s Hospital, Zhuhai Hospital Affiliated with Jinan University, Jinan University, Zhuhai, 519000 China

**Keywords:** Gastrointestinal cancer, Outcomes research

## Abstract

There is considerable potential for integrating transarterial chemoembolization (TACE), programmed death-(ligand)1 (PD-[L]1) inhibitors, and molecular targeted treatments (MTT) in hepatocellular carcinoma (HCC). It is necessary to investigate the therapeutic efficacy and safety of TACE combined with PD-(L)1 inhibitors and MTT in real-world situations. In this nationwide, retrospective, cohort study, 826 HCC patients receiving either TACE plus PD-(L)1 blockades and MTT (combination group, n = 376) or TACE monotherapy (monotherapy group, n = 450) were included from January 2018 to May 2021. The primary endpoint was progression-free survival (PFS) according to modified RECIST. The secondary outcomes included overall survival (OS), objective response rate (ORR), and safety. We performed propensity score matching approaches to reduce bias between two groups. After matching, 228 pairs were included with a predominantly advanced disease population. Median PFS in combination group was 9.5 months (95% confidence interval [CI], 8.4–11.0) versus 8.0 months (95% CI, 6.6–9.5) (adjusted hazard ratio [HR], 0.70, *P* = 0.002). OS and ORR were also significantly higher in combination group (median OS, 19.2 [16.1–27.3] vs. 15.7 months [13.0–20.2]; adjusted HR, 0.63, *P* = 0.001; ORR, 60.1% vs. 32.0%; *P* < 0.001). Grade 3/4 adverse events were observed at a rate of 15.8% and 7.5% in combination and monotherapy groups, respectively. Our results suggest that TACE plus PD-(L)1 blockades and MTT could significantly improve PFS, OS, and ORR versus TACE monotherapy for Chinese patients with predominantly advanced HCC in real-world practice, with an acceptable safety profile.

## Introduction

Hepatocellular carcinoma (HCC) is one of the most prevalent cancer and the leading cause of cancer-related death.^1^ Despite improved surveillance programs for HCC, around 80% of patients are diagnosed as intermediate or advanced stage disease.^2,3^ Transarterial chemoembolization (TACE) has become the standard of care for intermediate HCC globally, while it is also widely used in the advanced HCC.^3–9^ Systemic therapies, including molecular targeted therapies (MTT) and immunotherapies, are the standard treatment for advanced HCC in first-line setting.^10^

Sorafenib, lenvatinib are the tyrosine kinase inhibitors (TKIs) that once were approved as the first-line treatment for advanced HCC with limited survival benefits.^11–13^ Afterward, immunotherapies, including PD-1 and PD-L1 inhibitors, have shown promising efficacy and safety for advanced HCC in phase I and phase II trials.^14–16^ However, the phase III trials of CheckMate 459 and KEYNOTE-240 all failed to demonstrate superiority of anti-PD-1 therapy compared with standard of care.^17,18^ Recently, combination treatment with an anti-PD-(L)1 agent and anti-VEGF and/or TKIs has been proved to be effective and safe for advanced HCC by several RCTs and has been recommended in the first-line setting.^19–21^

Transarterial chemoembolization (TACE) results in necrosis of the tumor tissue and releases tumor antigens which may promote tumor-specific immune responses.^22^ TACE correlates with lower intra-tumoral exhausted effector cells (CD8^+^/PD-1^+^) and T regulatory cells (CD4^+^/FOXP3^+^) and may transform an immunosuppressive microenvironment into an immunosupportive setting to enhance the response of PD-(L)1 inhibitors.^23^ That provides the rationale with potential synergistic anti-tumor effect by combing TACE with PD-(L)1 inhibitors and MTT for both intermediate and advanced HCC.^24^ Based on such theory and encouraged by the success of RCTs for PD-(L)1 inhibitors combined with targeted agents, many clinical trials on TACE with PD-(L)1 treatment plus MTT are in progress to verify the potential synergetic effect.^25,26^ Unfortunately, none of such combination therapies with a large sample size have been reported by now.

Herein, the purpose of the CHANCE001 study was to describe the efficacy and safety in a nationwide, retrospective, propensity score matching (PSM) cohort of HCC patients who received TACE with PD-(L)1 inhibitors plus MTT versus TACE monotherapy in the real-world setting.

## Results

### Patient characteristics

**Fig. 1:**
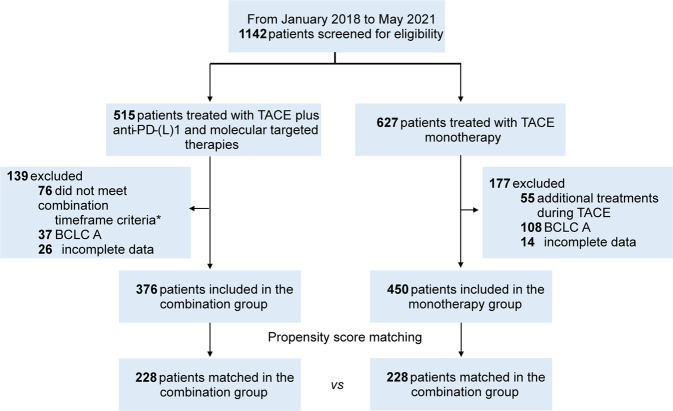
Flowchart of the study. *The criteria of combination timeframe were defined as administration of TACE concurrently with or up to 60 days before anti-PD-(L)1 blockades, and molecular targeted agents were concomitant with TACE or anti-PD-(L)1 blockades. *TACE* transarterial chemoembolization, *anti-PD-(L)1* anti-programmed death-(ligand)1, *BCLC* Barcelona Clinic Liver Cancer

There were 826 patients screened and included in this study, 376 of whom received TACE with PD-(L)1 blockades plus MTT, and 450 patients treated with TACE alone (Fig. 1). Prior to PSM, significantly higher tumor burden, worse liver function, and worse Eastern Cooperative Oncology Group (ECOG) performance status were observed in the combination group (Table 1). After matching in a 1:1 ratio, 456 patients remained (228 patients in each group) in the study cohorts, with a predominantly advanced disease population in both groups (65.8% and 66.2% in combination and monotherapy groups, respectively). There were no significant differences in baseline parameters between two groups (Table 1).

**Table 1:** Patient baseline characteristics of combination and monotherapy groups before and after PSM

Characteristics	Before PSM			After PSM		
	Combination group (*n* = 376)	Monotherapy group (*n* = 450)	*P-*value*	Combination group (*n* = 228)	Monotherapy group (*n* = 228)	*P*-value*
Median age (years)	55 (49–65)	61 (54–69)	<0.001	57 (50–67)	57 (50–66)	0.874
Sex			0.911			>0.999
Male	317 (84.3)	377 (83.8)		190 (83.3)	191 (83.8)	
Female	59 (15.7)	73 (16.2)		38 (16.7)	37 (16.2)	
Etiology			0.480			0.834
Hepatitis B virus	288 (76.6)	334 (74.2)		163 (71.5)	166 (72.8)	
Others	88 (23.4)	116 (25.8)		65 (28.5)	62 (27.2)	
Cirrhosis			<0.001			>0.999
Yes	281 (74.7)	279 (62.0)		166 (72.8)	166 (72.8)	
No	95 (25.3)	171 (38.0)		62 (27.2)	62 (27.2)	
Child-Pugh class			0.232			0.516
A	312 (83.0)	388 (86.2)		196 (86.0)	190 (83.3)	
B	64 (17.0)	62 (13.8)		32 (14.0)	38 (16.7)	
ECOG PS			<0.001			0.751
0	248 (66.0)	372 (82.7)		165 (72.4)	169 (74.1)	
1	128 (34.0)	78 (17.3)		63 (27.6)	59 (25.9)	
BCLC stage			<0.001			>0.999
B	103 (27.4)	226 (50.2)		78 (34.2)	77 (33.8)	
C	273 (72.6)	224 (49.8)		150 (65.8)	151 (66.2)	
Up-to-seven			0.662			0.913
≤7	91 (24.2)	102 (22.7)		55 (24.1)	57 (25.0)	
>7	285 (75.8)	348 (77.3)		173 (75.9)	171 (75.0)	
Macroscopic portal vein invasion			<0.001			0.778
Absent	192 (51.1)	293 (65.1)		124 (54.4)	120 (52.6)	
Present	184 (48.9)	157 (34.9)		104 (45.6)	108 (47.4)	
Extrahepatic spread			<0.001			>0.999
Absent	223 (59.3)	351 (78.0)		152 (66.7)	152 (66.7)	
Present	153 (40.7)	99 (22.0)		76 (33.3)	76 (33.3)	
TACE type			0.440			0.396
cTACE	279 (74.2)	322 (71.6)		163 (71.5)	172 (75.4)	
DEB-TACE	97 (25.8)	128 (28.4)		65 (28.5)	56 (24.6)	
HCC-related treatment history			<0.001			0.841
Absent	180 (47.9)	378 (84.0)		154 (67.5)	157 (68.9)	
Present	196 (52.1)	72 (16.0)		74 (32.5)	71 (31.1)	
Surgery	62 (16.5)	32 (7.1)	<0.001	27 (11.8)	31 (13.6)	0.674
TACE	165 (43.9)	46 (10.2)	<0.001	61 (26.8)	46 (20.2)	0.122
Ablation	35 (9.3)	18 (4.0)	0.002	14 (6.1)	18 (7.9)	0.583
Radiotherapy	38 (10.1)	8 (1.8)	<0.001	15 (6.6)	8 (3.5)	0.198

Data are median (interquartile range) or *n* (%)

*PSM* propensity score matching, *ECOG PS* Eastern Cooperative Oncology Group performance status, *BCLC* Barcelona Clinic Liver Cancer, *TACE* transarterial chemoembolization, *cTACE* conventional transarterial chemoembolization, *DEB-TACE* drug-eluting beads transarterial chemoembolization, *HCC* hepatocellular carcinoma

*Mann–Whitney U and the Student’s t test for continuous variables and Chi-squared test or Fisher exact test for categorical variables were applied

### Efficacy

**Fig. 2:**
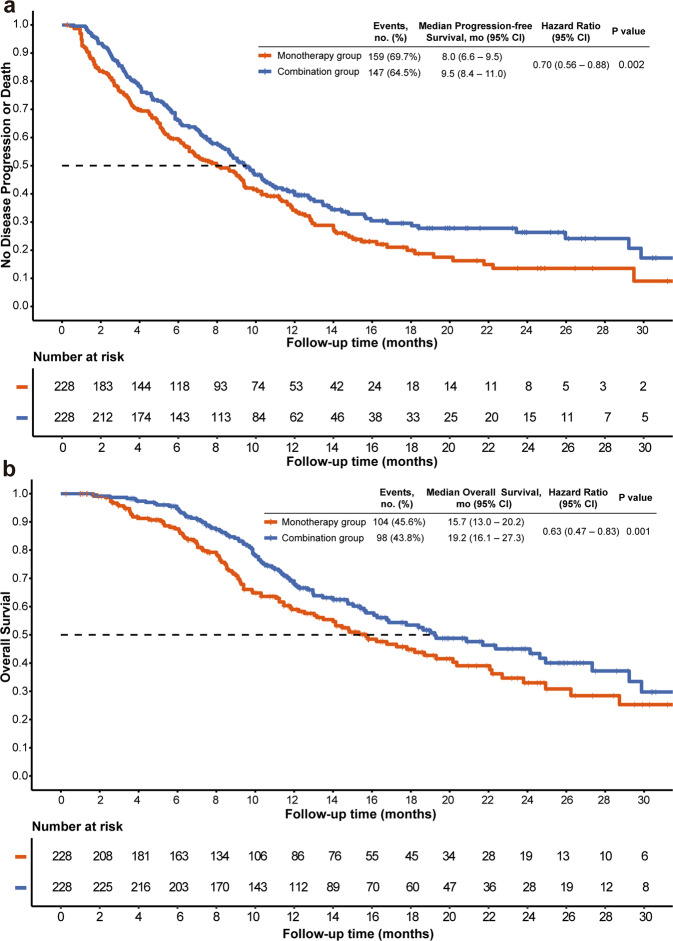
Kaplan–Meier analysis of progression-free survival (**a**) and overall survival (**b**)

**Fig. 3:**
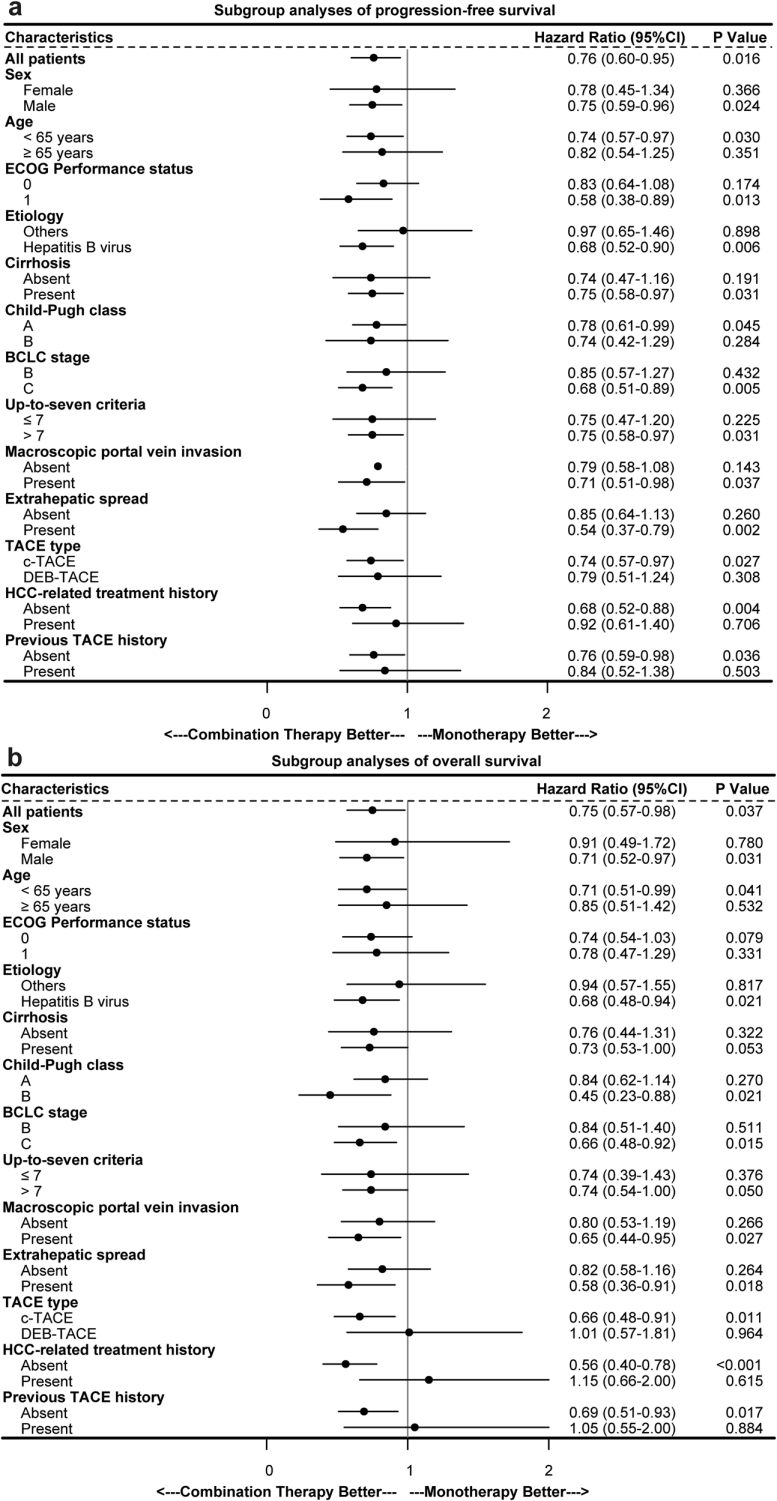
Subgroup analysis of progression-free survival (**a**) and overall survival (**b**). *HR* hazard ratio, *CI* confidence interval, *ECOG* Eastern Cooperative Oncology Group, *BCLC* Barcelona Clinic Liver Cancer, *TACE* transarterial chemoembolization, *cTACE* conventional transarterial chemoembolization, *DEB-TACE* drug-eluting beads transarterial chemoembolization, *HCC* hepatocellular carcinoma

After matching, the median follow-up time was 17.6 months (interquartile range [IQR], 15.0–20.2) in the combination group and 15.9 months (IQR, 15.0–18.6) in the monotherapy group (*P* = 0.200). During the follow-up, the median number of PD-(L)1 inhibitors and TACE in combination group were five cycles (IQR, 3–9 cycles) and two times (IQR, 1–4 times), respectively, and median number of TACE in monotherapy was three times (IQR, 2–6 times). 147 (64.5%) patients in the combination group and 159 (69.7%) in the monotherapy group had disease progression or died (Fig. 2). Median PFS was 9.5 months (95% CI, 8.4–11.0) in the combination group, which was significantly longer than that in the monotherapy group (8.0 months [95% CI, 6.6–9.5]; *P* = 0.015) (Fig. 2). A total of 98 (43.8%) patients in combination group and 104 (45.6%) in monotherapy group had died. There is a significant difference in terms of OS between combination group (median OS, 19.2 months; 95% CI, 16.1–27.3) and monotherapy group (median OS, 15.7 months; 95% CI, 13.0–20.2; *P* = 0.037). The higher ORR in the combination group was found (60.1% vs. 32.0%; *P* < 0.001). After adjusting the covariates, multivariate Cox proportional hazards models showed that combination therapy (for PFS, adjusted hazard ratio [HR], 0.70; 95% CI, 0.56–0.88; *P* = 0.002; for OS, HR, 0.63; 95% CI 0.47–0.83; *P* = 0.001; Table 2) was the independent positive prognostic indicator for PFS and OS in the matched cohorts. The sensitivity analyses supported these findings. Subgroup analyses showed that the combination group had a trend persisted on better PFS and OS benefits compared to the monotherapy group (Fig. 3).

**Table 2:** Predictors of progression-free survival and overall survival after matching

	Univariable analysis			Multivariable analysis		
	HR	95% CI	*P*-value	HR	95% CI	*P*-value
*PFS analyses*						
ECOG PS (1 *vs*. 0)	1.10	0.85–1.41	0.466			
Etiology (HBV *vs*. others)	0.92	0.72–1.18	0.532			
Cirrhosis (present *vs*. absent)	1.05	0.81–1.36	0.706			
Child-Pugh class (B *vs*. A)	1.27	0.94–1.72	0.123			
BCLC stage (C *vs*. B)	1.75	1.37–2.23	<0.001	1.50	0.96–2.34	0.073
Up-to-seven criteria (>7 *vs*. ≤7)	1.42	1.09–1.86	0.010	1.27	0.95–1.69	0.103
Macroscopic portal vein invasion (present *vs*. absent)	1.47	1.17–1.85	0.001	1.01	0.71–1.45	0.939
Extrahepatic spread (present *vs*. absent)	1.46	1.15–1.85	0.002	1.16	0.84–1.61	0.360
TACE type (DEB-TACE *vs*. cTACE)	1.06	0.82–1.36	0.677			
HCC-related treatment history (present *vs*. absent)	0.63	0.49–0.81	<0.001	0.76	0.48–1.19	0.224
Previous TACE history (present *vs*. absent)	0.66	0.50–0.87	0.004	0.95	0.59–1.54	0.837
Treatment (combination therapy *vs*. monotherapy)	0.76	0.60–0.95	0.016	0.70	0.56–0.88	0.002
*OS analyses*						
ECOG PS (1 *vs*. 0)	1.12	0.83–1.52	0.461			
Etiology (HBV *vs*. others)	0.87	0.64–1.17	0.345			
Cirrhosis (present *vs*. absent)	1.05	0.76–1.44	0.765			
Child-Pugh class (B *vs*. A)	1.56	1.10–2.21	0.013	1.21	0.84–1.74	0.307
BCLC stage (C *vs*. B)	1.84	1.36–2.49	<0.001	1.14	0.66–1.98	0.631
Up-to-seven criteria (>7 *vs*. ≤7)	1.81	1.27–2.59	0.001	1.49	1.03–2.17	0.034
Macroscopic portal vein invasion (present *vs*. absent)	1.75	1.33–2.31	<0.001	1.32	0.84–2.05	0.225
Extrahepatic spread (present *vs*. absent)	1.49	1.12–1.98	0.007	1.29	0.88–1.89	0.200
TACE type (DEB-TACE *vs*. cTACE)	1.08	0.79–1.49	0.621			
HCC-related treatment history (present *vs*. absent)	0.46	0.34–0.63	<0.001	0.54	0.30–0.96	0.037
Previous TACE history (present *vs*. absent)	0.54	0.38–0.77	0.001	1.05	0.56–1.96	0.881
Treatment (combination therapy *vs*. monotherapy)	0.75	0.57–0.98	0.037	0.63	0.47–0.83	0.001

The multivariable analysis includes the variables with *P*-value ≤0.1 from the univariable analysis

*HR* hazard ratio, *CI* confidence intervals, *ECOG PS* Eastern Cooperative Oncology Group performance status, *HBV* hepatitis B virus, *BCLC* Barcelona Clinic Liver Cancer, *TACE* transarterial chemoembolization, *cTACE* conventional TACE, *DEB-TACE* drug-eluting beads TACE, *HCC* hepatocellular carcinoma

### Safety

After matching, the AEs were reported by 134 of 228 patients (58.8%) in the combination group and 101 (44.3%) in the monotherapy group (Table 3 & Supplementary Table 1). Grade 3 or 4 AEs occurred in 36 patients (15.8%) in the combination group and 17 (7.5%) in the monotherapy group. No grade 5 AEs were observed in either matching group. In the combination group, PD-(L)1 inhibitors were discontinued due to AEs in 13 (5.7%) patients. Molecular targeted agents were discontinued in 27 (11.8%) patients because of AEs. There were 9 patients (3.9%) who experienced dose interruption of anti-PD-(L)1 agents in consequence of AEs. AEs resulting in dose reduction or interruption of TKIs and/or anti-VEGF agents were reported by 22 (9.6%) patients.

**Table 3:** Adverse events from any cause after PSM

Variable	Combination group (*n* = 228)	Monotherapy group (*n* = 228)
Patients with an adverse event from any cause	134 (58.8)	101 (44.3)
Grade 1 or 2 event^a^	98 (43.0)	84 (36.8)
Grade 3 event^a^	33 (14.5)	17 (7.5)
Grade 4 event^a^	3 (1.3)	0
Grade 5 event^a^	0	0
Discontinuation of anti-PD-(L)1 therapies	13 (5.7)	N/A
Discontinuation of molecular targeted therapies	27 (11.8)	N/A
Dose interruption of anti-PD-(L)1 therapies	9 (3.9)	N/A
Dose reduction or interruption of molecular targeted therapies	22 (9.6)	N/A

*PSM* propensity score matching, *PD-1* programmed death 1, *PD-L1* programmed death ligand 1, *N/A* not applicable

Data are n (%)

^a^Numbers represent the highest grades assigned

In the monotherapy group, AEs of any grade (>10%) were abdominal pain (30.3%), elevated aspartate aminotransferase (18.0%), nausea (16.7%), elevated alanine aminotransferase (14.9%), and vomiting (12.3%) (Supplementary Table 2). In the combination group, AEs of any grade (>10%) were elevated aspartate aminotransferase (47.8%), abdominal pain (39.0%), elevated alanine transaminase (36.8%), pyrexia (25.4%), elevated bilirubin (22.4%), hypertension (13.6%), hand-foot skin reaction (13.2%), and proteinuria (10.5%) (Supplementary Table 3).

## Discussion

The present multicenter, retrospective, matched cohort study (CHANCE001) based on the nationwide data showed that TACE with PD-(L)1 inhibitors plus MTT significantly improved PFS, OS, and ORR in predominantly advanced HCC patients when compared to TACE alone. Subgroup analyses showed generally consistent survival benefits across clinical subgroups. The incidence of AEs in combination group appears to be slightly higher than that reported in monotherapy, and most AEs were easily managed with mild-to-moderate severity.

Worse liver function and performance status were observed in the present study compared with previously reported IMbrave 150 and ORIENT-32 trials.^20,21^ Longer PFS (9.5 [95% CI 8.4–11.0] *vs*. 6.9 [95% CI 5.7–8.6] *vs*. 4.6 [95% CI 4.1–5.7] months) with higher ORR (60.1% *vs*. 33.2% *vs*. 24.3%, according to modified Response Evaluation Criteria in Solid Tumors [mRECIST]) were achieved in our study than those in the IMbrave 150 and ORIENT-32 trials, respectively. Median OS was 19.2 months [95% CI 16.1–27.3] in the CHANCE001 combination group with poorer clinical characteristics, which was similar to updated IMbrave 150 trial data (19.2 months [95% CI 17.0–23.7]).^27^

The efficacy benefits of TACE with PD-(L)1 inhibitors plus MTT were generally consistent across clinical subgroups, including those of relevance to HCC (ECOG performance status, HBV etiology, baseline tumor burden, and the with or without macrovascular invasion and/or extrahepatic spread). Besides, early combination therapy is expected to lead to greater potential gain than those who had previous treatment history. As these were subgroup analyses, these findings should be interpreted with caution. All AEs from any cause were reported in 98.2% and 99% of patients receiving PD-(L)1 inhibitors plus MTT, while grade ≥3 AEs were 61.6% and 56% in the IMbrave 150 and ORIENT-32 trials, respectively.^20,21^ Most AEs were mild-to-moderate severity and readily managed or reversible in our study, with a small percentage of patients discontinuing PD-(L)1 inhibitors (5.7%) or molecular targeted agents (11.8%) due to AEs.

There are rationales for the combination of TACE with PD-(L)1 inhibitors plus MTT.^28,29^ First, TACE induces hypoxia microenvironment and VEGF elevation expression in the residual surviving cancerous tissue.^30^ Antiangiogenic therapy (antibodies targeting VEGF or TKIs) might delay the revascularization and recurrence of tumor after TACE.^28^ Unfortunately, several RCTs, including SPACE, Post-TACE, and TACE-2 trials, failed to demonstrate the expected results in HCC patients who received TACE plus sorafenib.^31^ The possible causes of the failures of these reported trials mainly involved population selection, endpoint selection, combination strategies, and timing and duration of drug administration.^32,33^ Second, the liver contains immunosuppressive cells and has an intrinsic immune tolerance, which may decrease the immune response to tumor.^34^ TACE can induce the release of tumor antigens and proinflammatory cytokines and is associated with reduced intra-tumoral exhausted effector cells and T regulatory cells.^22,28^ Thus, it can locoregionally induce immunogenic cell death in HCC and turn the immunosuppressive “cold tumor” into an immunogenic “hot tumor” by restoring the immune microenvironment to further improve immune response.^23,35,36^ Third, there is a close relationship between angiogenesis and suppression of anti-tumor immunity. The VEGF, a key regulator factor of tumor angiogenesis, can directly influence immune cells and facilitate immune evasion, and indirectly influence immunity by increasing vessel permeability.^37^ For example, VEGF can result in an immunosuppressive tumor microenvironment by hindering the maturation and function of dendritic cells and increasing T regulatory cells and myeloid-derived suppressor cells recruitments.^38^ Targeting VEGF can restore anti-tumor activity and enhance the efficacy of immune checkpoint inhibitors.^37,38^

There are several limitations in the present study. The retrospective nature may introduce the risk of selection bias, as demonstrated by the difference in baseline characteristics. To minimize the effects of this limitation, we performed the PSM and several sensitivity analyses which are frequently used in real-world studies. Second, various kinds of PD-(L)1 inhibitors and MTT were provided by several esteemed or new pharmaceutical companies in the study. Notably, all the PD-(L)1 inhibitors and MTT applied in the present study are recommended for HCC either in the west or in Chinese guidelines. We pool all drugs together because they have similar targets inspired by the notion of “umbrella” trials to identify the efficacy of different drugs, based on different mutations in one cancer.^39^ This design can validate a treatment strategy involving a mixture of agents and has been successfully conducted in several trials.^40,41^ Along with increasing sample size in the database, stratified analyses could identify better combination protocols in the future. Third, bias due to allocation constraints of immunotherapy caused by financial resources and routine follow-up examinations disrupted by COVID-19 could influence the results of the study.

In conclusion, compare to TACE monotherapy, TACE with anti-PD-(L)1 plus MTT shows significantly better PFS, OS, and ORR for chinese patients with predominantly advanced HCC in a real-world setting, with an acceptable safety profile. Before the outcomes of ongoing RCTs are reported, the present study provides proper evidence for this combination therapy strategy in HCC.

## Materials and methods

### Patient criteria

Applicable Institutional Review Boards at all participating hospitals reviewed and approved this retrospective multicenter study and written informed consent was waived due to its retrospective nature. The study was performed in accordance with the Declaration of Helsinki. The study was registered with ClinicalTrials.gov, NCT04975932, and is reported as per the STROBE statement for observational cohort studies. Patients with HCC who received either TACE with PD-(L)1 inhibitors plus MTT (TKI or anti-VEGF monoclonal antibody) (combination group) or TACE alone (monotherapy group) at 59 academic hospitals in China from January 2018 to May 2021 were screened. The diagnosis of HCC was confirmed according to the guidelines.^5,6^ The data for this study were derived from the database of the national registry platform entitled “Chinese Liver Cancer Clinical Study Alliance (CHANCE)” sponsored by the Chinese College of Interventionalists. All of the included patients were not previously reported and were not enrolled in those industry-sponsored clinical trials.

Inclusion criteria were: (1) histologically or clinically confirmed diagnosis of HCC with Barcelona Clinic Liver Cancer (BCLC) stage B or C; (2) Child-Pugh grade A/B without presence of uncontrollable ascites or hepatic encephalopathy; (3) ECOG performance status 0 or 1; (4) received combination of TACE with PD-(L)1 blockades and MTT or TACE monotherapy during the same period. The criteria of combination timeframe were defined as administration of TACE concurrently with or up to 60 days before anti-PD-(L)1 therapy, and molecular targeted agents were concomitant with TACE or anti-PD-(L)1 therapy. At least one cycle of anti-PD-(L)1 agent should be used after the TACE procedure. Patients with HCC-related treatment histories such as surgery, ablation, TACE, or radiotherapy were also included. Patients with incomplete clinical or follow-up information were excluded.

The decision-making for the treatment using TACE alone or TACE combined with systemic agents according to BCLC guidelines or China National Liver Cancer guidelines for HCC,^9,10^ financial burden, physicians’ favor, and patients’ selection. In some of the participating hospitals, multidisciplinary teams for HCC dominated it. Generally, physicians would let the patient and his/her family members know the advantages and disadvantages of TACE with or without PD-(L)1 inhibitors and molecular targeted agents, including potential therapeutic effects, adverse events (AEs), and costs before the decision-making.

### Transarterial chemoembolization procedure

All patients underwent standardized conventional TACE (cTACE) or drug-eluting beads TACE (DEB-TACE) procedures.^9,42^ “On-demand” TACE was repeated based on the evidence of viable tumors or intra-hepatic recurrence by contrast-enhanced computed tomography (CT) or magnetic resonance imaging (MRI). The whole TACE procedures were carried out by physicians with at least 10 years of experience in interventional radiology from participating centers. TACE was discontinued if one of the following conditions occurred: (1) deterioration in hepatic function to Child-Pugh C (uncontrollable ascites, severe jaundice, overt hepatic encephalopathy, or hepatorenal syndrome); (2) ECOG performance status >2; (3) continuous progression of target lesions after three TACE sessions according to the clinical practice of the participating centers.

### Anti-PD-(L)1 agents administration

For patients receiving TACE with PD-(L)1 inhibitors and MTT, several PD-(L)1 inhibitors including atezolizumab, pembrolizumab, nivolumab, camrelizumab, sintilimab, tislelizumab, and toripalimab were used based on the guidelines and availability in China (Supplementary Table 4). All the PD-(L)1 inhibitors were administrated based on their standard dose and frequency. Concurrent anti-PD-(L)1 therapy was administrated at least three days before or after TACE. Dose reduction was not allowed but interrupted PD-(L)1 inhibitors because of AEs were allowed. Patients received anti-PD-(L)1 agents until disease progression or unacceptable toxic effects.

### Molecular targeted agents administration

For patients receiving TACE combined with PD-(L)1 inhibitors and MTT, several molecular targeted (TKI or anti-VEGF) agents including sorafenib, lenvatinib, donafenib, regorafenib, apatinib, anlotinib, and bevacizumab were administrated with their standard dose (Supplementary Table 4).^11,13,20,43–45^ All the oral agents were administrated within two weeks before or after TACE or anti-PD-(L)1 agents, and bevacizumab was administrated along with anti-PD-(L)1 agents. Dose reduction because of grade 3 or 4 AEs was allowed except for bevacizumab. Patients received molecular targeted agents until disease progression or unacceptable toxic effects.

### Follow-up and assessments

Patient assessments were arranged before every treatment session (both for TACE and anti-PD-(L)1 therapy) or during every routine follow-up at a minimum of 3–4 weeks intervals (supplementary Fig. 1). At each visit, all AEs were recorded and assessed per the National Cancer Institute Common Terminology Criteria for Adverse Events version 5.0, and standard laboratory investigations (complete blood count, chemistry, coagulation panel, and urinalysis) completed. Patients received contrast-enhanced CT/MRI follow-up at a 6–9 weeks interval. Tumor response evaluation was conducted by two independent radiologists with more than five years of experience at each center according to mRECIST. All the radiologists who participated in the study received lecture-based and online instruction training that focus on the standardized tumor response. Patients were followed up routinely until death or the end of the study (May 30, 2022).

### Outcomes

The primary outcome was PFS, defined as time from the initiation of TACE procedure to first tumor progression or death from any cause. For patients treated with anti-PD-(L)1 inhibitors before TACE, PFS was defined as the period from the initiation of anti-PD-(L)1 therapy to first tumor progression or death from any cause. Secondary outcomes included OS (the time from the initiation of combination therapies or TACE monotherapy to death from any cause), ORR (percentage of patients with a confirmed complete or partial response), and safety.

### Statistical analysis

To address the imbalance of potential confounders between two groups, PSM analysis was performed using 1:1 nearest-neighbor method without replacement using caliper widths of 0.05. Propensity scores were estimated using a logistic regression model by including the following variables: sex, age, ECOG performance status, hepatitis B virus (HBV), cirrhosis, Child-Pugh grade, up-to-seven criteria, BCLC stage, portal vein invasion, extrahepatic spread, and HCC-related treatment history. The standardized mean difference was used to evaluate the covariate balance for the propensity-matched cohorts (supplementary Fig. 2).

Sample size calculation were performed using PASS. The patient characteristics were summarized using median with IQR for continuous variables, and using frequencies with proportions for categorical variables. Student’s t-test or Mann–Whitney U test was used to analyze continuous variables. Chi-squared test or Fisher exact test was applied to analyze categorical variables. The difference in PFS and OS between the two groups were compared with the use of a log-rank test. The survival curves were plotted by the method of Kaplan–Meier. Cox proportional hazards models were used for univariable and multivariable analyses by the forward procedure on the propensity-matched sample. Forest plots were used to display these data.

A subgroup analysis comparing PFS between combination group and monotherapy group was planned for prespecified subgroups, including ECOG performance status, BCLC stage, up-to-seven criteria, cirrhosis, HBV, Child-Pugh grade, portal vein invasion, extrahepatic spread, TACE type, previous TACE history, and HCC-related treatment history. To assess the robustness of the PSM analysis, sensitivity analysis was conducted by using different matching methods and factors. The between-group difference in PFS was also assessed by multivariable Cox proportional hazards model on unmatched all patients. To reduce the potential confounding factors, we performed the inverse probability of treatment weighting analysis to adjust the covariates of the multivariable Cox model on unmatched all patients for sensitivity analysis.

A two-tailed *P-*value of <0.05 was considered statistically significant. All the above statistical analyses were performed using R (version 4.1.0; R Project for Statistical Computing, http://www.r-project.org), and SPSS (version 24.0; IBM, Somers, NY).

## Supplementary information

Revised Supplementary Materials

## Data Availability

Correspondence and reasonable requests for original dataset should be addressed to Dr. Gao-Jun Teng (gjteng@seu.edu.cn).
