# Supplementary material for: Transarterial chemoembolization with PD-(L)1 inhibitors plus molecular targeted therapies for hepatocellular carcinoma (CHANCE001)
Source: Signal Transduct Target Ther. 2023 Feb 8;8:58. doi: 10.1038/s41392-022-01235-0 (PMC9905571; doi:10.1038/s41392-022-01235-0)
Supplement: Supplementary file 1 — Revised Supplementary Materials [file 41392_2022_1235_MOESM1_ESM.docx]

Supplementary Materials for

Transarterial chemoembolization plus anti-PD-(L)1 and molecular targeted therapies for hepatocellular carcinoma (CHANCE001)

Hai-Dong Zhu, Hai-Liang Li, Ming-Sheng Huang, Wei-Zhu Yang, Guo-Wen Yin, Bin-Yan Zhong, Jun-Hui Sun, Zhi-Cheng Jin, Jian-Jian Chen, Nai-Jian Ge, Wen-Bin Ding, Wen-Hui Li, Jin-Hua Huang, Wei Mu, Shan-Zhi Gu, Jia-Ping Li, Hui Zhao, Shu-Wei Wen, Yan-Ming Lei, Yu-Sheng Song, Chun-Wang Yuan, Wei-Dong Wang, Ming Huang, Wei Zhao, Jian-Bing Wu, Song Wang, Xu Zhu, Jian-Jun Han, Wei-Xin Ren, Zai-Ming Lu, Wen-Ge Xing, Yong Fan, Hai-Lan Lin, Zi-Shu Zhang, Guo-Hui Xu, Wen-Hao Hu, Qiang Tu, Hong-Ying Su, Chuan-Sheng Zheng, Yong Chen, Xu-Ya Zhao, Zhu-Ting Fang, Qi Wang, Jin-Wei Zhao, Ai-Bing Xu, Jian Xu, Qing-Hua Wu, Huan-Zhang Niu, Jian Wang, Feng Dai, Dui-Ping Feng, Qing-Dong Li, Rong-Shu Shi, Jia-Rui Li, Guang Yang, Hai-Bin Shi, Jian-Song Ji, Yu-E Liu, Zheng Cai, Po Yang, Yang Zhao, Xiao-Li Zhu, Li-Gong Lu, and Gao-Jun Teng

Correspondence to: Gao-Jun Teng (gjteng@seu.edu.cn) or Li-Gong Lu (luligong1969@126.com) or Xiao-Li Zhu (zhuxiaoli90@163.com)

**This PDF file includes:**

Materials and Methods

Supplementary Figs. 1 to 2

Supplementary Tables 1 to 4

Reference

Materials and Methods

**Detailed transarterial chemoembolization procedure protocol**

Patients included in the study received conventional transarterial chemoembolization (cTACE) or drug-eluting beads TACE (DEB-TACE). All the TACE procedures were applied according to standardization protocols in the all-participated hospital1-3. Adequate visualization of all tumor-feeding feeding arteries should be obtained during the procedure, including vessels’ origin, variant anatomy, and ectopic or collateral blood supply. Feeding arteries of the tumors were as selective as possible in order to obtain better treatment efficacy and to reduce treatment-related complications both for cTACE and DEB-TACE. The endpoint of TACE is defined as a “tree in winter” appearance in case of non-selective TACE.

For cTACE, an emulsion of mixtures of lipiodol (2–20 ml) and chemotherapeutic drugs was injected to the feeding arteries of the tumors. Doxorubicin is the most common single chemotherapeutic drug. The dosage of chemotherapeutic drug used could be body surface area-based, liver function-based, weight-based, or even empiric. Chemotherapeutic drugs including doxorubicin (10–100 mg), epirubicin (5–120 mg), oxaliplatin (100–200 mg), cisplatin (10–100 mg) and other drugs were selected according to clinical practice of the participating centers. The ethiodized oil and chemotherapeutic drugs should be mixed into an emulsion and configured as a “water-in-oil” emulsifier to improve its stability. The volume ratio of ethiodized oil to drug aqueous solution is usually 2:1. The volume of ethiodized oil injected is generally determined by the size and vascularity of the tumor, with common usage of 5–15 mL. Finally, particulate embolic agents (e.g., standardized gelatin sponge particles, microspheres, polyvinyl alcohol particles) should be were used following embolization with ethiodized oil chemoembolic emulsion to achieve a satisfied embolization endpoint.

For DEB-TACE, a dose of 2–4 ml DC beads (Biocompatibles, Farnham, United Kingdom) or Callispheres beads (Jiangsu Hengrui Medicine Co., Ltd., Jiangsu, China) or HepaSpheres beads (Biosphere Medical, Inc., South Jordan, UT) with a diameter of 100–300 or 300–500 μm loaded with epirubicin (with a maximum dose of 100 mg) were introduced. Additional embolization was applied if satisfied embolization endpoint was not achieved.

“On demand” TACE procedures were repeated based on the demonstration of viable tumors or intrahepatic recurrences by contrast-enhanced computed tomography (CT) or magnetic resonance imaging (MRI). All the TACE procedures were performed by physicians with at least 10 years of experience on interventional radiology from participating centers. When residual viable tumors were confirmed or new lesions developed in patients with adequate liver function, repeated TACE was performed.

TACE was discontinued if one of the following conditions occurred: 1) deterioration of liver function to Child-Pugh C (uncontrollable ascites, severe jaundice, overt hepatic encephalopathy, or hepatorenal syndrome); 2) Eastern Cooperative Oncology Group (ECOG > 2); 3) continued progression of target lesions after 3 TACE sessions according to clinical practice of the participating centers.

**Sensitivity analyses**

Propensity score matching was performed by a different method. We used one to one optimal matching method without replacement. Propensity scores were calculated using a logistic regression model by the following variables: sex, age, ECOG performance status, hepatitis B virus, cirrhosis, Child-Pugh grade, up-to-seven criteria, Barcelona Clinic Liver Cancer (BCLC) stage, portal vein invasion, extrahepatic spread, and hepatocellular carcinoma (HCC)-related treatment history. After matching, 752 patients remained in the study cohorts (376 patients in each group). There were still differences that remained for baseline characteristics between the two groups. The median progression-free survival (PFS) was 8.7 months (95% CI, 7.9–9.9) in the combination group, which was significantly longer than that in the monotherapy group (8.2 months [95% CI, 7.1–9.3]; P = 0.03). The median overall survival (OS) was 19.2 months (95% CI, 16.9–22.9) with an objective response rate (ORR) of 52.4% in the combination group, which was significantly longer than that in the monotherapy group (15.8 months [95% CI, 14.1–18.7]; P = 0.02; ORR, 37.8%, P < 0.001).

Then, we performed propensity score matching with several key clinical factors (ECOG performance status, hepatitis B virus, Child-Pugh grade, and BCLC stage), which indicated patients’ general well-being, etiology, liver function, and tumor stage. On the other hand, these excluded factors (sex, age, cirrhosis, portal vein invasion, extrahepatic spread, and HCC-related treatment history) were either balanced before matching, or not definitive prognostic for PFS in the previous study, or associated with the above four factors. The 1:1 nearest-neighbor method with caliper widths of 0.05 was used. After matching, 610 patients remained in the study cohorts (305 patients in each group). There were small differences remained for other characteristics not included in the propensity score matching. The median PFS was 9.5 months (95% CI, 8.6–10.9) in the combination group, which was significantly longer than that in the monotherapy group (8.2 months [95% CI, 6.8–9.4]; P = 0.002). The median OS was 19.5 months (95% CI, 16.9–24.9) with an ORR of 57.4% in the combination group, which was significantly longer than that in the monotherapy group (15.7 months [95% CI, 13.6–20.4]; P = 0.02; ORR, 34.8%, P < 0.001).

For the inverse probability of treatment weighting analysis, we calculated the probability of receiving the combination therapy (propensity score) for each patient using a logistic regression model. The model included the following variables: sex, age, ECOG performance status, hepatitis B virus, cirrhosis, Child-Pugh grade, up-to-seven criteria, BCLC stage, portal vein invasion, extrahepatic spread, and HCC-related treatment history. We calculated individual weights using the propensity score as follows: 1/propensity score for patients receiving the combination therapy, and 1/ (1–propensity score) for monotherapy. After adjusted the covariates, multivariate Cox regression analysis showed that combination therapy (for PFS, hazard ratio [HR] 0.76; 95% CI 0.62–0.92; P = 0.006; for OS, HR 0.73; 95% CI 0.57–0.94; P = 0.013) were the independent prognostic indicators in all patients weighting analysis cohort.

**Sample size calculation**

For sample size calculation, the median PFS for TACE monotherapy was set as 7 months according to AASLD Consensus Conference^1^, and the median PFS for combination group was set as 10 months according to comprehensive evaluation based on investigator opinion and previous study^2-4^. Follow-up time was set as 12 months. The proportion dropping out of each group was 0.02 per year. A two-sided log-rank test with an overall sample size of 396 patients (198 in each group) achieves 80% power at a 0.05 significance level. Above sample size calculation were performed using PASS (version 15.0.5).


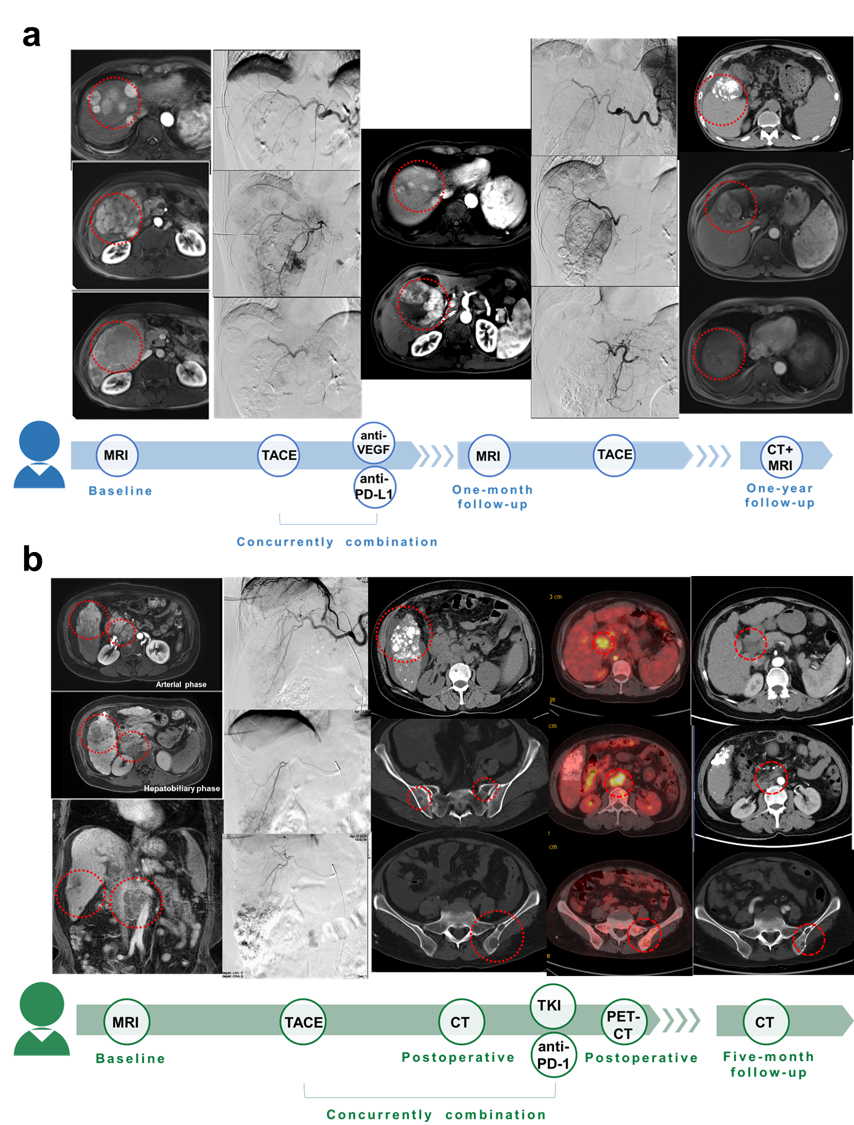


**Supplementary Fig. 1** Images in two cases with HCC treated with TACE plus anti-PD-(L)1 and molecular targeted therapies. a. A 57-year-old man had a history of chronic hepatitis B ≥ 20 years. The baseline MRI showed massive HCC with peritumoral star lesions (red dotted circle). There is no vascular invasion or extrahepatic spread. The patient was diagnosed as BCLC-B stage and received TACE combined with atezolizumab (1200 mg, ivgtt, q3w) and bevacizumab (900 mg, ivgtt, q3w). The first follow-up MRI showed the patient had stable disease. Then, a second TACE was performed. Radiologic partial response was achieved thereafter. The patient is still being followed up (> 24 months). b. A 59-year-old woman had a history of chronic hepatitis B ≥ 30 years with baseline serum alpha-fetoprotein > 10000 ng/mL. Baseline MRI showed HCC lesions in the right liver lobe with enlargement of multiple porta hepatic and retroperitoneal lymph nodes (red dotted circle). Then, the patient was diagnosed as BCLC-C stage and received an initial TACE procedure. The postoperative CT and positron emission tomography (PET)-CT also show active metastases in pelvic and lymph nodes. The patient concurrently received tislelizumab (200 mg, ivgtt, q3w) and sorafenib (200 mg, po, bid). Partial response was achieved and shown on the subsequent follow-up imaging. This patient has long-term sustained efficacy and is still alive (> 24 months).

**
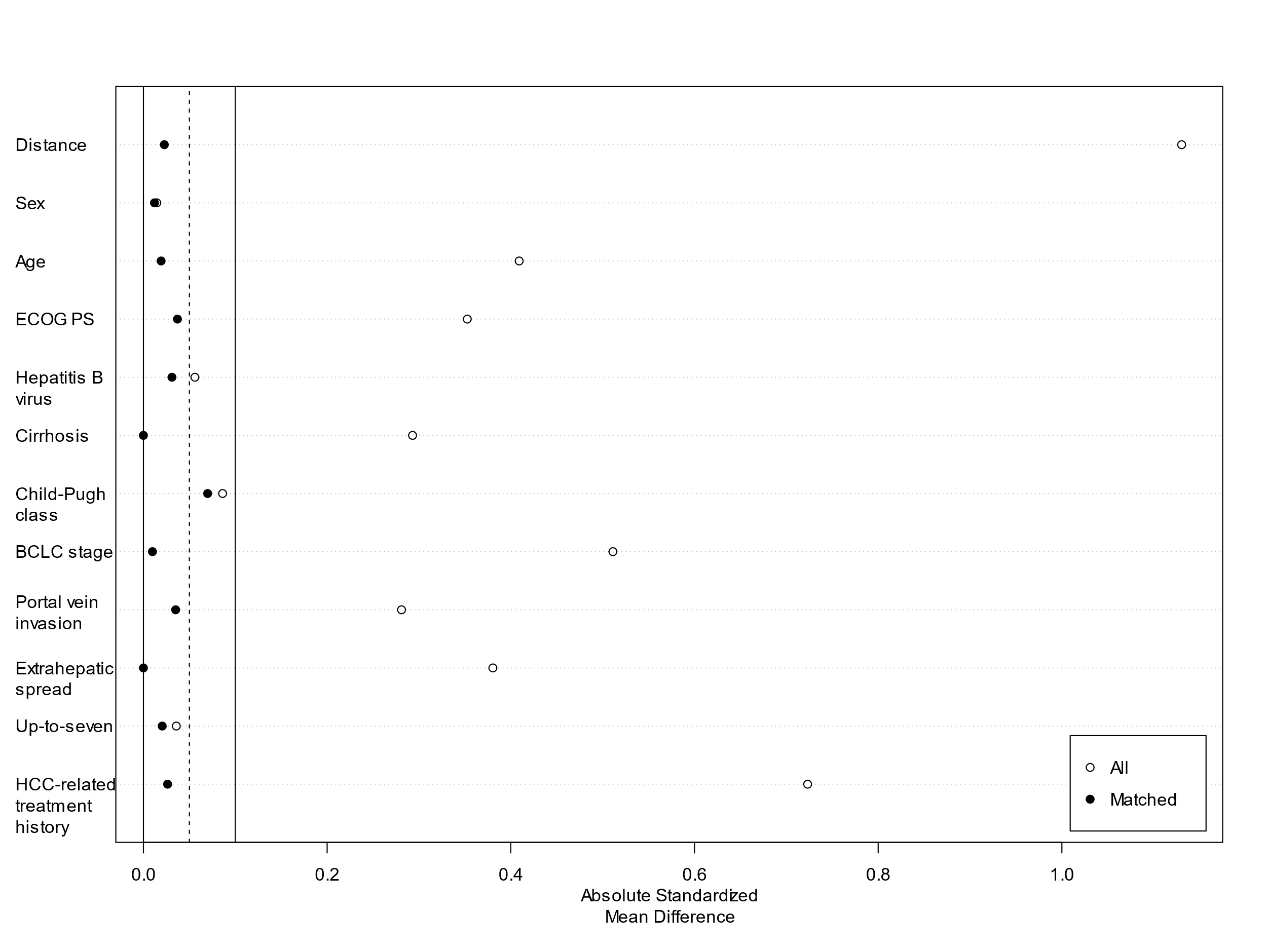
**

Supplementary Fig. 2 Absolute standardized mean difference of propensity score matching

Supplementary Table 1. Treatment-related adverse events after matching

| Variable | Combination group  (n = 228) | Monotherapy group  (n = 228) |
| --- | --- | --- |
| TACE-related event | 93 (40.8) | 96 (42.1) |
| Grade 1 or 2 event^*^ | 83 (36.4) | 82 (36.0) |
| Grade 3 event^*^ | 9 (3.9) | 14 (6.1) |
| Grade 4 event^*^ | 1 (0.4) | 0 |
| Anti-PD-(L)1 therapies-related event | 69 (30.3) | N/A |
| Grade 1 or 2 event^*^ | 56 (24.6) | N/A |
| Grade 3 event^*^ | 12 (5.3) | N/A |
| Grade 4 event^*^ | 1 (0.4) | N/A |
| Molecular targeted therapies-related event | 67 (29.4) | N/A |
| Grade 1 or 2 event^*^ | 46 (20.2) | N/A |
| Grade 3 event^*^ | 20 (8.8) | N/A |
| Grade 4 event^*^ | 1 (0.4) | N/A |

*PD-1* programmed death 1, *PD-L1* programmed death ligand 1, *N/A* not applicable

Data are n (%). ^*^Numbers represent the highest grades assigned

Supplementary Table 2. Adverse events in monotherapy group after matching

| Adverse events | All grades | Grade 1 or 2 | Grade 3 | Grade 4 | Grade 5 |
| --- | --- | --- | --- | --- | --- |
| Abdominal pain | 69 (30.3) | 66 (28.9) | 3 (1.3) | 0 | 0 |
| Increased AST | 41 (18.0) | 36 (15.8) | 5 (2.2) | 0 | 0 |
| Nausea | 38 (16.7) | 37 (16.2) | 1 (0.4) | 0 | 0 |
| Increased ALT | 34 (14.9) | 30 (13.2) | 4 (1.8) | 0 | 0 |
| Vomiting | 28 (12.3) | 27 (11.8) | 1 (0.4) | 0 | 0 |
| Elevated bilirubin | 24 (10.5) | 20 (8.8) | 4 (1.8) | 0 | 0 |
| Pyrexia | 21 (9.2) | 18 (7.9) | 3 (1.3) | 0 | 0 |
| Fatigue | 9 (3.9) | 9 (3.9) | 0 | 0 | 0 |
| Anorexia | 8 (3.5) | 7 (3.1) | 1 (0.4) | 0 | 0 |

*AST* aspartate aminotransferase, *ALT* alanine aminotransferase

Data are n (%). The number of patients in monotherapy group after matching is 228

| Adverse events | All grades | Grades 1 or 2 | Grade 3 | Grade 4 | Grade 5 |
| --- | --- | --- | --- | --- | --- |
| Increased AST | 109 (47.8) | 103 (45.2) | 6 (2.6) | 0 | 0 |
| Abdominal pain | 89 (39.0) | 86 (37.7) | 3 (1.3) | 0 | 0 |
| Increased ALT | 84 (36.8) | 80 (35.1) | 4 (1.8) | 0 | 0 |
| Pyrexia | 58 (25.4) | 53 (23.2) | 4 (1.8) | 1 (0.4) | 0 |
| Elevated bilirubin | 51 (22.4) | 50 (21.9) | 1 (0.4) | 0 | 0 |
| Hypertension | 31 (13.6) | 25 (11.0) | 6 (2.6) | 0 | 0 |
| HFSR | 30 (13.2) | 24 (10.5) | 6 (2.6) | 0 | 0 |
| Proteinuria | 24 (10.5) | 18 (7.9) | 6 (2.6) | 0 | 0 |
| Fatigue | 21 (9.2) | 15 (6.6) | 6 (2.6) | 0 | 0 |
| Vomiting | 21 (9.2) | 21 (9.2) | 0 | 0 | 0 |
| Nausea | 21 (9.2) | 21 (9.2) | 0 | 0 | 0 |
| Hypothyroidism | 20 (8.8) | 20 (8.8) | 0 | 0 | 0 |
| RCCEP | 14 (6.1) | 14 (6.1) | 0 | 0 | 0 |
| Rash | 13 (5.7) | 12 (5.3) | 0 | 1 (0.4) | 0 |
| Diarrhea | 12 (5.3) | 9 (3.9) | 3 (1.3) | 0 | 0 |
| Thrombocytopenia | 7 (3.1) | 5 (2.2) | 1 (0.4) | 1 (0.4) | 0 |
| Pruritus | 5 (2.2) | 5 (2.2) | 0 | 0 | 0 |
| Hepatitis | 3 (1.3) | 2 (0.9) | 1 (0.4) | 0 | 0 |
| Infusion related reaction | 3 (1.3) | 3 (1.3) | 0 | 0 | 0 |
| Pneumonitis | 2 (0.9) | 0 | 2 (0.9) | 0 | 0 |
| Enterocolitis | 2 (0.9) | 2 (0.9) | 0 | 0 | 0 |
| Decreased WBC count | 2 (0.9) | 2 (0.9) | 0 | 0 | 0 |
| Neutropenia | 1 (0.4) | 1 (0.4) | 0 | 0 | 0 |
| hyperglycemia | 1 (0.4) | 0 | 1 (0.4) | 0 | 0 |
| Thoracic hemorrhage | 1 (0.4) | 0 | 1 (0.4) | 0 | 0 |
| Alopecia | 1 (0.4) | 1 (0.4) | 0 | 0 | 0 |
| Glomerulonephritis | 1 (0.4) | 1 (0.4) | 0 | 0 | 0 |
| Hyperthyroidism | 1 (0.4) | 1 (0.4) | 0 | 0 | 0 |
| Hypophysis | 1 (0.4) | 0 | 1 (0.4) | 0 | 0 |
| Elevated serum amylase | 1 (0.4) | 1 (0.4) | 0 | 0 | 0 |

Supplementary Table 3. Adverse events in combination group after matching

*AST* aspartate aminotransferase, *ALT* alanine aminotransferase, *HFSR* hand-foot skin reaction, *RCCEP* reactive cutaneous capillary endothelial proliferation, *WBC* white blood cell count

Data are n (%). The number of patients in combination group after matching is 228

Supplementary Table 4. Agents administration protocol

| **Agents** | **Targets** | **Administration** |
| --- | --- | --- |
| **Anti-PD-(L)1 agents administration** | | |
| Atezolizumab, Tecentriq®, F. Hoffmann-La Roche AG, Basel, Switzerland | PD-L1 | 1200 mg, ivgtt, q3w |
| Camrelizumab, AiRuiKa®, Jiangsu Hengrui Medicine Co. Ltd, Suzhou, China | PD-1 | 200 mg or 3mg/kg, ivgtt, q3w |
| Nivolumab, Opdivo®, Bristol-Myers Squibb Holdings Pharma, Ltd.Liability Company, New York, U.S.A. | PD-1 | 3 mg/kg, ivgtt, q2w |
| Pembrolizumab, Keytruda®, Merck Sharp& Dohme Corp., Kenilworth, N.J., U.S.A. | PD-1 | 200 mg, ivgtt, q3w |
| Sintilimab, Tyvyt®, Innovent Biologics, Inc., Suzhou, China | PD-1 | 200 mg, ivgtt, q3w |
| Tislelizumab, Baize’an®, BeiGene Ltd., Beijing, China | PD-1 | 200 mg, ivgtt, q3w |
| Toripalimab, Tuoyi®, Junshi Bioscience Co., Ltd, Shanghai, China | PD-1 | 240 mg, ivgtt, q3w |
| **Molecular targeted agents administration** | | |
| Apatinib, Aitan®, Jiangsu Hengrui Medicine Co. Ltd, Lianyungang, China | VEGFR2 | 250 mg, po, qd |
| Anlotinib, Fukewei®,  Chia Tai Tianqing Pharmaceutical Group Co., Ltd, Lianyungang, China | VEGFR1–VEGFR3, FGFR1–FGFR4, PDGFR, KIT receptor | 8 mg, po, qd |
| Bevacizumab, Avastin®, F. Hoffmann-La Roche AG, Basel, Switzerland | VEGFA | 15 mg/kg, ivgtt, q3w |
| Donafenib, Zepsun®, Suzhou Zelgen Biopharmaceuticals Co, Ltd., Suzhou, China | VEGFR, PDGFR, Raf/MEK/ERK kinase | 200 mg, po, bid |
| Lenvatinib, Lenvanix®, Eisai Inc., Japan | VEGFR1–VEGFR3, PDGFR, FGFR1–FGFR4, RET | 8 mg, po, qd (for bodyweight < 60 kg) or 12 mg, po, qd (for bodyweight ≥ 60 kg) |
| Regorafenib, Stivarga®, Bayer HealthCare Pharmaceuticals Inc. | VEGFR1–VEGFR3,  PDGFR, RAF kinase, FGFR1, FGFR2 | 160 mg, po, qd |
| Sorafenib, Nexavar®, Bayer AG Kaiser-Wilhelm-Allee, Leverkusen, Germany | VEGFR1–VEGFR3,  PDGFR, RAF kinase, KIT receptor | 400 mg, po, bid |

**REFERENCE**

1 Llovet, J. M. *et al.* Trial Design and Endpoints in Hepatocellular Carcinoma: AASLD Consensus Conference. *Hepatology*. **73 Suppl 1**, 158-191 (2021).

2 Finn, R. S. *et al.* Atezolizumab plus Bevacizumab in Unresectable Hepatocellular Carcinoma. *N. Engl. J. Med.* **382**, 1894-1905 (2020).

3 Zheng, L. *et al.* Efficacy and Safety of TACE Combined With Sorafenib Plus Immune Checkpoint Inhibitors for the Treatment of Intermediate and Advanced TACE-Refractory Hepatocellular Carcinoma: A Retrospective Study. *Front. Mol. Biosci*. **7**, 609322 (2020).

4 Llovet, J. M. *et al.* Locoregional therapies in the era of molecular and immune treatments for hepatocellular carcinoma. *Nat. Rev. Gastroenterol. Hepatol.* **18**, 293-313 (2021).
